# Supplementary material for: A new trajectory approach for investigating the association between an environmental or occupational exposure over lifetime and the risk of chronic disease: Application to smoking, asbestos, and lung cancer
Source: PLoS One. 2020 Aug 12;15(8):e0236736. doi: 10.1371/journal.pone.0236736 (PMC7423115; doi:10.1371/journal.pone.0236736)
Supplement: S1 File — (PDF) [file pone.0236736.s001.pdf]

Supplementary Materials

Table of contents

Table of contents 1
Numerical values used in the JEM (Table S1) 2
Distribution of exposure intensities (Fig S1) 3
Individual observed lifetime trajectories (Fig S2) 4
Equations of the latent class mixed models 5
Description of included/excluded subjects for the statistical analysis (Table S2) 6
Discrimination capacity of the two LCMM (Table S3, Table S4) 7
Results in current smokers (Fig S3, Table S5) 8
Association between asbestos and smoking classifications (Table S6) 11
R code 12

Numerical values used in the JEM (Table S1)

Table S1. Numerical Values of Probability, Frequency, and Intensity of Asbestos Exposure Used in the Job Exposure Matrix (JEM) to Derive Individual Average Annual Daily Intensity of Exposure.

Asbestos exposure characteristics	Definition	Numerical values used to calculate annual doses		
Probability of exposure (% of workers exposed)				
Non exposed	0	0		
Possible	> 0 - 5	0.025		
Probable	5 - 30	0.175		
Likely	30 - 70	0.5		
Definite	≥ 70	0.85		
Frequency of exposure (% of work time)				
Sporadic	> 0-5	0.025		
Occasional	5-30	0.175		
Frequent	30-70	0.5		
Continuous	≥ 70	0.85		
Intensity of exposure (equivalent fibres/ml)*		Passive exposure	Indirect exposure	Direct exposure
Very low	> 0 - 0.01	0.0005	0.0025	0.005
Low	0.01 - 0.1	0.005	0.025	0.05
Medium	0.1 - 1	0.05	0.25	0.5
High	1 - 10	0.5	2.5	5
Very high	≥ 10	2	10	15

* Intensity of exposure was defined as a combination of the intensity of exposure due to specific task and work environment contamination. Asbestos JEM was based on expert judgment, and intensity of exposure was expressed in equivalent fibres/ml. Three types of exposure were defined: Passive exposure (workers were exposed according to diffuse contamination of buildings); indirect exposure (workers were exposed by other workers using asbestos materials); direct exposure (workers used directly asbestos materials).

Distribution of exposure intensities (Fig S1)

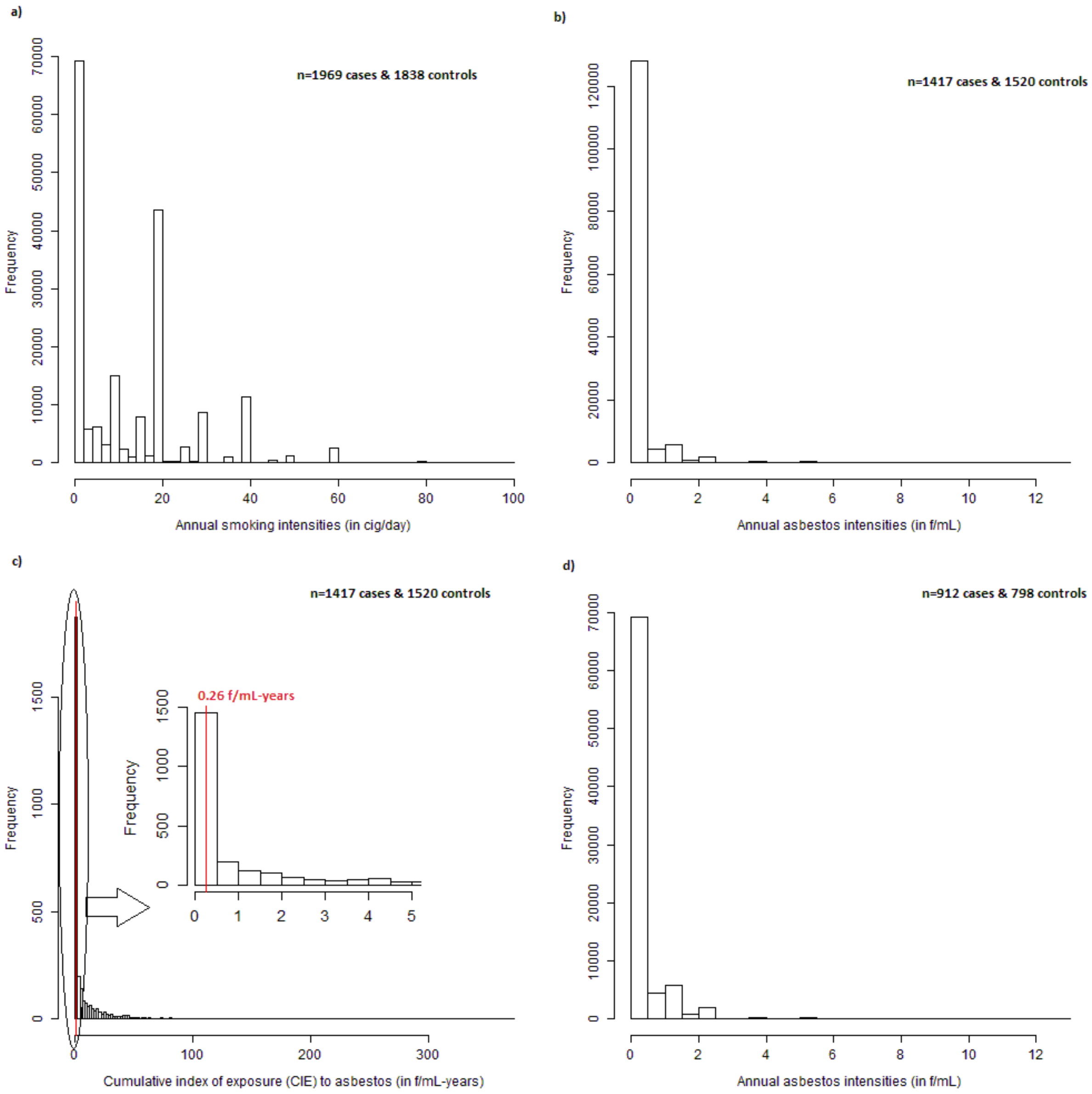

Fig S1. Distribution of exposure. On Panel a), distribution of annual average intensities of smoking (number of cigarettes smoked per day) in all ever smokers. On Panel b), distribution of annual average intensities of occupational exposure to asbestos (in f/mL) in all subjects ever occupationally exposed to asbestos. On Panel c), distribution of the cumulative index of exposure (in f/mL-years) at the index date in all subjects ever occupationally exposed to asbestos, with a focus on low cumulative exposure. On Panel d), distribution of annual average intensities of occupational exposure to asbestos (in f/mL) in subjects who had cumulated more than 0.26 f/mL-years over lifetime. ICARE case-control study, 2001-2007, France

Individual observed lifetime trajectories (Fig S2)

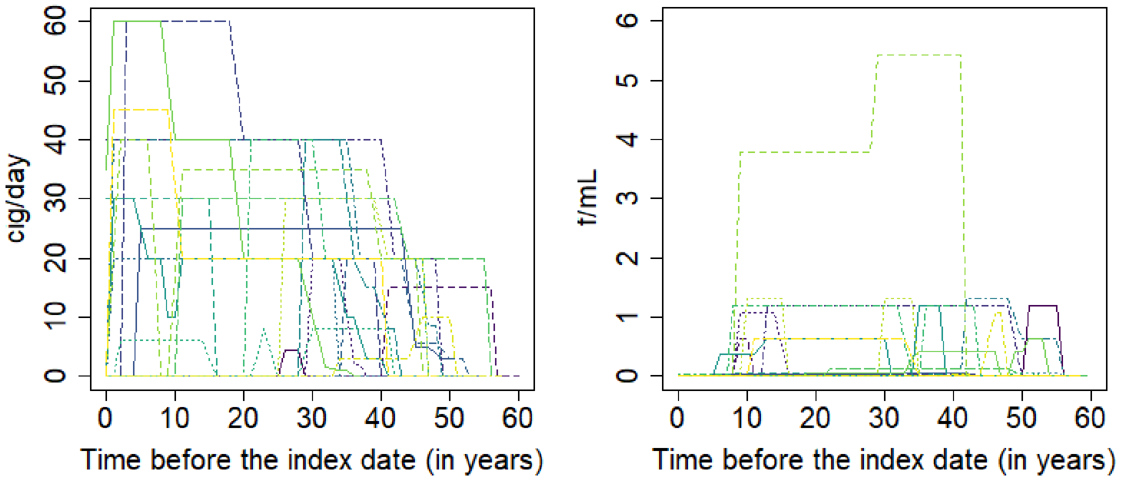

Fig S2. 20 Random Observed Individual Trajectories. On the left panel, for smoking intensities, and on the right panel, for intensities of occupational exposure to asbestos. ICARE Case-Control Study, 2001-2007, France

Equations of the latent class mixed models

Two separate LCMM were used to identify classes of smoking trajectories in all ever smokers, and classes of asbestos exposure trajectories in subjects who accumulated more than 0.26f/mL-years over at the index date. Each LCMM was made of two sub-models whose equations are described below.

Sub-model 1: multinomial logistic regression for latent class membership

The probability that a subject i belongs to the latent class g (g = 1, …, G) was given by:

$$\pi_{ig} = P(c_i = g) = \frac{e^{\gamma_{0g}}}{\sum_{l=1}^{G} e^{\gamma_{0l}}}, \qquad \text{(Equation 1)}$$

where c_i denotes a discrete random variable which equals g if the subject i belongs to latent class g, and γ_{0g} is the intercept for class g. For identifiability γ_{0G}=0.

Sub-model 2: class-specific mixed model

The observed annual intensity of subject i (i= 1, .., n) in the jth year (j = 0,…, n_i) before diagnosis/interview, Y_{ij}, was modelled using a latent process mixed model, that is a linear mixed model adapted to non-Gaussian continuous variables. More specifically, sub-model 2 simultaneously normalized Y_{ij} with a parameterized link function H, and modeled its trajectory with a spline function of time:

$$H(Y_{ij})|_{c_i=g} = \left(b_{0g} + u_{0ig} 1_{t_{ij} \in Hist_i}\right) + \sum_l b_{lg} B_l(t_{ij}) + \varepsilon_{ij} \qquad \text{(Equation 2)}$$

where

- H was an I-splines function to account for non-normality of annual intensities with 3 manual knots at 0, 20 and 100 cig/day for smoking and 0, 0.05, 12.6 f/mL for asbestos
- t_{ij} was the jth year before the index date for subject i.
- ε_{ij} were assumed to be independent Gaussian measurement errors with variance σ^2_{ε}.
- b_{0g} andb_{lg} were class-specific fixed effects.
- $B_l(t)$ were the splines basis function of time before index date with 3 inner knots placed at quartiles (12, 24 and 36 years).
- $1_{t_{ij} \in Hist_i}$ was an indicator which equaled one if the time t_{ij} occurred during the exposure history of subject i ($Hist_i$), 0 otherwise.
- u_{0ig} was the intercept class-specific random effect. We assumed u_{0ig} N(0, $w_g^2\sigma^2_u$), where σ^2_u was an unspecified common variance and w_g a coefficient allowing for class-specific variability.

Description of included/excluded subjects for the statistical analysis (Table S2)

Table S2. Characteristics of included/excluded subjects for the statistical analysis. ICARE Case-Control Study, 2001-2007, France.

	Cases				Controls			
	Included (n=2026)		Excluded for incomplete data on history of exposure (n=250)		Included (n=2610)		Excluded for incomplete data on history of exposure (n=170)	
**Age at index date (n, mean (sd))**	2026	60.3 (9.0)	250	60.4 (9.6)	2610	58.2 (9.9)	170	56.1 (9.9)
**Area of residence (n,%)**								
Calvados	240	11.8	32	12.8	336	12.9	22	12.9
Doubs-Territoire de Belfort	103	5.1	3	1.2	109	4.1	3	1.8
Hérault	227	11.2	25	10.0	343	13.1	17	10.0
Isère	346	17.1	25	10.0	375	14.4	32	18.8
Loire Atlantique	255	12.6	18	7.2	297	11.4	14	8.2
Manche	225	11.1	37	14.8	22	8.5	25	14.7
Bas-Rhin	247	12.2	55	22.0	331	12.7	29	17.1
Haut-Rhin	53	2.63	3	1.2	88	3.4	1	0.6
Somme	224	11.1	45	18.0	365	14.0	22	12.9
Vendée	106	5.2	7	2.8	144	5.5	5	2.9
**Education level (n, %)**								
Elementary school or less	600	29.6	75	30.0	489	18.7	32	18.8
Middle school	779	38.5	90	36.0	1028	39.4	53	31.2
High school	177	8.7	8	3.2	293	11.2	17	10.0
University	253	12.5	20	8.0	693	26.6	59	34.7
Other	21	1.0	4	1.6	18	0.7	1	0.6
Missing	196	9.7	53	21.2	89	3.4	8	4.7

Discrimination capacity of the two LCMM (Table S3, Table S4)

From the estimated LCMM, we derived the estimated posterior probability for each subject to belong to each latent class given his exposure data. Each subject was then a posteriori classified in the class where he had the highest probability to belong. We further derived the posterior classification table where, for each latent class, we calculated the mean posterior probability to belong to each latent class among subjects a posteriori classified in the given class. For example for smoking, 873 subjects had their highest probability to belong to Class 2, and were thus a posteriori classified in this class (Table S3). Their mean probability to belong to Class 2 was 0.9694, while their mean probability to belong to Class 1 was 0.0292 only. Overall, the model has a good discrimination capacity if diagonal terms are close to 1 and all others close to 0.

Table S3. Posterior Classification Table for the Four Identified Latent Classes of Smoking Intensities. ICARE Case-Control Study, 2001-2007, France.

	N*	Mean of the posterior probabilities of belonging to each class			
		1	2	3	4
Class 1	1985	0.9832	0.0130	0.0029	0.0009
Class 2	873	0.0292	0.9694	0.0013	0.0000
Class 3	483	0.0083	0.0010	0.9871	0.0037
Class 4	466	0.0032	0.0000	0.0035	0.9933

*Number of subjects a posteriori classified in the class

Table S4. Posterior Classification Table for the Four Identified Latent Classes of Occupational Asbestos Intensities. ICARE Case-Control Study, 2001-2007, France.

	N*	Mean of the posterior probabilities of belonging to each class			
		1	2	3	4
Class 1	914	0.9776	0.0047	0.0101	0.0075
Class 2	227	0.0195	0.9697	0.0108	0.0000
Class 3	348	0.0258	0.0061	0.9670	0.0011
Class 4	221	0.0319	0.0000	0.0037	0.9644

*Number of subjects a posteriori classified in the class

Results in current smokers (Fig S3, Table S5)

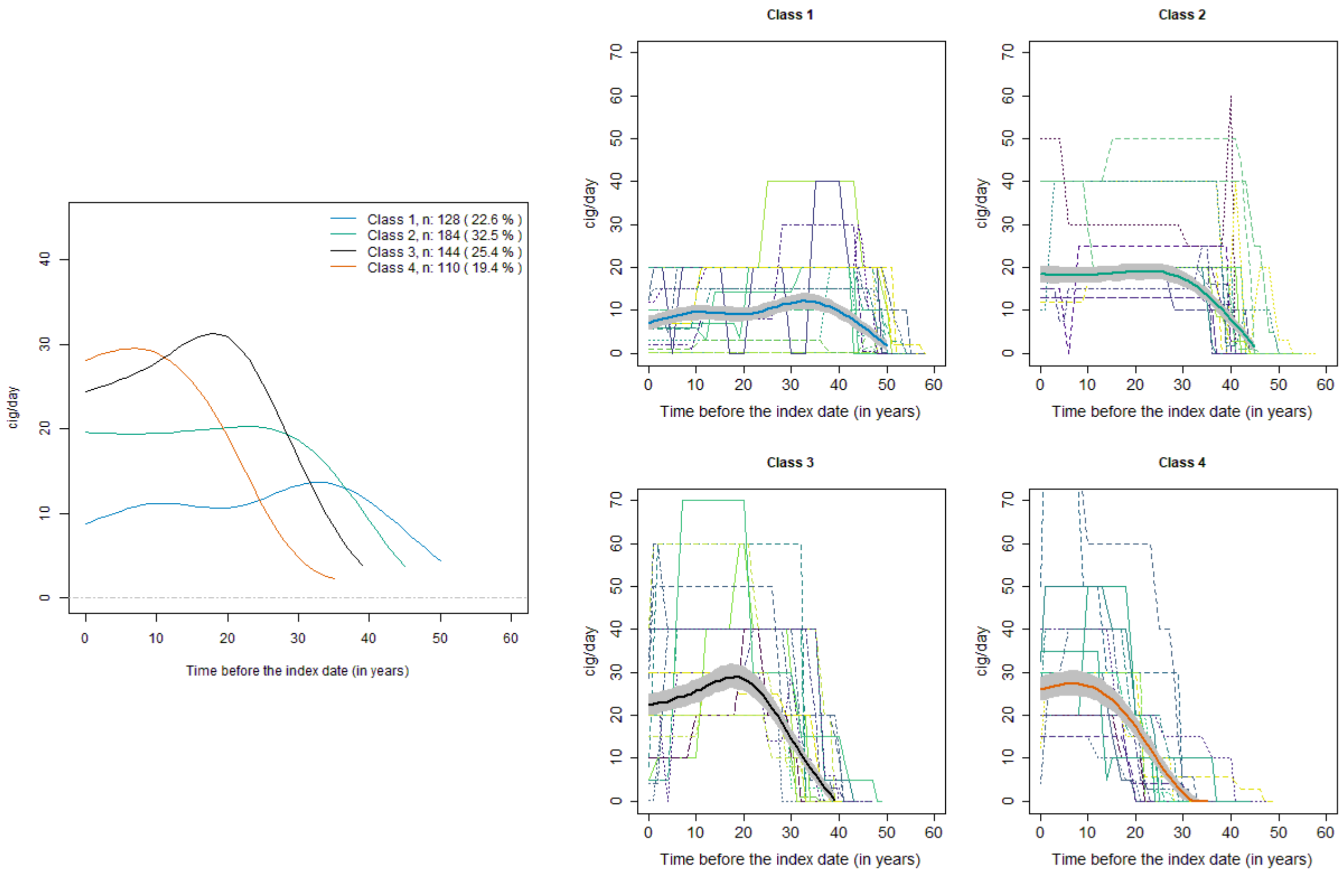

Fig S3. Lifetime Trajectories of Smoking Intensities in current smokers only, ICARE Case-Control Study, 2001-2007, France. The left panel shows the estimated mean trajectory of smoking intensity in the four latent classes. The right panel shows for each class, 20 randomly selected observed individual trajectories of subjects who had a high probability (close to 1) to belong to the class, with the bold line representing the estimated mean trajectory in the Class, with its 95% CI.

Table S5. Association Between Trajectories of Smoking Intensity in current smokers and Lung Cancer, ICARE Case-Control Study, 2001-2007, France.

Trajectory of smoking exposure	Number of cases and controls[a]	Age at index date (years) median (5th-95th percentile)	Cigarettes-years[b] median (5th-95th percentile)	Smoking duration (years)[c] median (5th-95th percentile)	Average smoking intensity[d] (cig/day) median (5th-95th percentile)	Age at initiation (years) median (5th-95th percentile)	OR[e] (95%CI)	OR[f] (95%CI)	OR[g] (95%CI)
Never smokers	57						1.00	1.00	1.00
	772								
Ex smokers	1606	61	524	32	18	17	13.4	13.0	13.1
	1635	(44-74)	(22-1520)	(6-52)	(3-37)	(13-23)	(10.1, 17.8)	(9.8, 17.2)	(11.1, 15.5)
Class 1	73	63	630	48	13	16	17.6	16.6	16.9
	55	(49-70)	(30-1467)	(25-56)	(1-28)	(12-21)	(11.2, 27.5)	(10.6, 26.1)	(12.9, 22.1)
Class 2	123	57	732	41	18	17	30.3	29.6	29.3
	61	(49-66)	(220-1593)	(31-50)	(7-34)	(12-24)	(20.1, 45.7)	(19.6, 44.7)	(23.0, 37.4)
Class 3	95	51	702	35	21	16	39.5	37.7	37.7
	49	(41-59)	(212-2008)	(26-43)	(8-50)	(12-22)	(25.1, 62.1)	(23.9, 59.5)	(28.8, 49.4)
Class 4	72	44	540	27	20	17	50.3	48.3	49.4
	38	(34-58)	(202-1602)	(18-40)	(9-48)	(14-25)	(30.3, 83.5)	(29.0, 80.4)	(36.6, 66.9)
AIC							5537	5463	5472

OR: odds ratio; CI: confidence interval

a From a posteriori classification for Classes 1 to 4

b Sum of all annual intensities

c Total effective duration of smoking over all periods of smoking, excluding periods of interruptions

d Average intensity over all periods of smoking

e Adjusted for age at the index date in years (second-degree fractional polynomial with powers (-2,-2)) and area of residence (*département*)

f Adjusted for age at the index date in years (second-degree fractional polynomial with powers (-2,-2)), area of residence (*département*), and cumulative index of occupational exposure to asbestos in f/mL-years (first-degree fractional polynomial with power 0)

g Adjusted for age at the index date in years (second-degree fractional polynomial with powers (-2,-2)), area of residence (*département*), and asbestos exposure trajectory class membership.

Association between asbestos and smoking classifications (Table S6)

Table S6. Cross-tabulation between the classes of asbestos exposure and smoking.

Asbestos / Smoking	Never Exposed	Class 1 : Constant moderate intensity	Class 2 : Recent high intensity	Class 3 : Distant high intensity	Class 4 : Very distant moderate intensity	Class 5 : Low cumulative exposures
Never smokers	350 (20.6)	133 (14.6)	26 (11.5)	46 (13.2)	26 (11.8)	248 (20.2)
Class 1 : Constant moderate intensity	733 (43.1)	361 (39.5)	107 (47.1)	171 (49.1)	92 (41.6)	521 (42.5)
Class 2 : Recent high intensity	276 (16.2)	178 (19.5)	81 (35.7)	82 (23.6)	46 (20.8)	210 (17.1)
Class 3 : Long term very high intensity	171 (10.1)	129 (14.1)	11 (4.8)	29 (8.3)	26 (11.8)	117 (9.5)
Class 4 : Distant very high intensity	169 (9.9)	113 (12.4)	2 (0.9)	20 (5.7)	31 (14.0)	131 (10.7)
Total	1699 (100)	914 (100)	227 (100)	348 (100)	221 (100)	1227 (100)

R code

```
#once installed, packages to load
library(lcmm)
library(splines)
library(epiDisplay)

#data basis with one ligne per individual repeated measure of exposure
Base_Tab<-read.csv2("BaseIcare.csv",header=TRUE,sep=";")


######################################
#latent class mixed model for 1 class
mod1.lcmm<-lcmm(fixed=doseTab_10~ns(t_TSI,knots=c(12,24,36),Boundary=c(0,64)),
        random=~-1+Ind_IntEa,subject="numid",ng=1,link="3-manual-splines",intnodes=c(2),
        data=Base_Tab,maxiter=50)
######
#fixed effets part of mixed model
######
#fixed=doseTab_10~ns(t_TSI,knots=c(12,24,36),Boundary=c(0,64))
#
#-doseTab_10: repeated measures of exposure intensities (here, smoking in cig/day)
#-t_TSI : time variable corresponds to the used time axis, here the time before index date
#-ns() for considering a restricted cubic splines of time with 3 inner knots located at quartiles of
considered time
#(12,24 and 36 years before index date)

######
#random effets part of mixed model
######
#random=~-1+Ind_IntEa
#-Ind_IntEa : specific random variable which equals to 1 if the year of t_TSI is during the subject's
history exposure


######
#I-splines transformation for the repeated measures
######
#link="3-manual-splines": for a I-spline at 3 knots with one interior node being entered in the
argument intnodes  positionné par l'utilisateur
#here intnodes equals to 2 which corresponds to  20 cig/day since the repeated measures were
divided by 10 to avoid potential numerical estimation issues


######
#other arguments
######
```

```
#subject="numid" : name of variable to identify each subject

#ng=1 : number of latent classes

#maxiter: maximum number of iterations for the optmization algorithm

#######################################

#######################################
#gridsearch to estimate a model with g>1 latent classes from random initial values derived from an estimated model with 1 latent class
m4_Tab.lcmm<-
gridsearch(rep=50,maxiter=30,minit=mod1.lcmm,lcmm(fixed=doseTab_10~ns(t_TSI,knots=c(12,24,36),Boundary=c(0,64)),random=~-1+Ind_IntEa,subject="numid",
                                    mixture=~ns(t_TSI,knots=c(12,24,36),Boundary=c(0,64)),
                                    ng=4,link="3-manual-splines",intnodes=c(2),
                                    data=Base_Tab,nwg=TRUE))

#arguments of the function:
#rep= the number of models to estimate from different random initial values
#maxiter= the number of iterations for the optmization algorithm
#minit= the model with one class latent for the generation of random initial values
#last argument corresponds to the model to estimate with ng=4 for a model with 4 latent classes with proportional variance (nwg=TRUE)

#######################################


#posterior classification from the estimated model and the associated posterior classification table
postprob(m4_Tab.lcmm)


#######################################
#Mean predicted trajectories

#a new profile to estimate these mean predicted trajectories of the identified classes
datnew<-
data.frame(t_TSI=seq(min(Base_Tab$t_TSI),max(Base_Tab$t_TSI),by=1),Ind_IntEa=c(rep(1,max(Base_Tab$t_TSI)-min(Base_Tab$t_TSI)+1)))

#2 different methods to calculate these predictions, methInteg=0 (by default) for Gaussian Hermite integration
pred_GH50<- predictY(m4_Tab.lcmm, newdata=datnew, var.time="t_TSI", draws=TRUE,nsim=50)

#methInteg=1 for Monte carlo integration, slower but better with a required relatively important number of points (nsim)
```

```
pred_MC<- predictY(m4_Tab.lcmm, newdata=datnew, var.time="t_TSI", draws=TRUE,methInteg =
1,nsim=200)
########################################

########################################
#plot of predicted mean trajectories

#number and percentage of subjects classified a posteriori in each class
n_cl1<-length(which(m4_Tab.lcmm$pprob$class==1))
p_cl1<-round(n_cl1/length(unique(Base_Tab$numid)),3)*100
n_cl2<-length(which(m4_Tab.lcmm$pprob$class==2))
p_cl2<-round(n_cl2/length(unique(Base_Tab$numid)),3)*100
n_cl3<-length(which(m4_Tab.lcmm$pprob$class==3))
p_cl3<-round(n_cl3/length(unique(Base_Tab$numid)),3)*100
n_cl4<-length(which(m4_Tab.lcmm$pprob$class==4))
p_cl4<-round(n_cl4/length(unique(Base_Tab$numid)),3)*100


#to get the 95th percentile of time axis observed for each class among the subjects a posteriori
classified into that class
Max_Cl1<-quantile(Base_Tab$t_TSI[Base_Tab$Class==1],probs=c(0.95))
Max_Cl2<-quantile(Base_Tab$t_TSI[Base_Tab$Class==2],probs=c(0.95))
Max_Cl3<-quantile(Base_Tab$t_TSI[Base_Tab$Class==3],probs=c(0.95))
Max_Cl4<-quantile(Base_Tab$t_TSI[Base_Tab$Class==4],probs=c(0.95))


plot(0:Max_Cl1,pred_MC$pred[1:(Max_Cl1+1),1]*10,ylim=c(0,45),xlim=c(0,60),legend=NULL,col="blu
e",lty=1,type="l",ylab="cig/day",xlab="Time before the index date (in years)")
lines(0:Max_Cl2,pred_MC$pred[1:(Max_Cl2+1),2]*10,legend=NULL,col="green",lty=1)
lines(0:Max_Cl3,pred_MC$pred[1:(Max_Cl3+1),3]*10,legend=NULL,col="black",lty=1)
lines(0:Max_Cl4,pred_MC$pred[1:(Max_Cl4+1),4]*10,legend=NULL,col="red",lty=1)
abline(h=0,col="grey",lty=4)
legend(x="topright",bty="n",ncol=1,lty=c(1,1),col=c("blue","green","black","red"),legend=c(paste("Cl
ass 1, n:",n_cl1,"(",p_cl1,"% )"),
                                       paste("Class 2, n:",n_cl2,"(",p_cl2,"% )"),
                                       paste("Class 3, n:",n_cl3,"(",p_cl3,"% )"),
                                       paste("Class 4, n:",n_cl4,"(",p_cl4,"% )")))
########################################

########################################
##logistic regression models with the classification variable of smoking exposure Cl_Tab and the
case-control status kt

#Base_All : duplicated database with 4 rows for ever smokers and 1 row for never smokers
#WeiTab : estimated probabilities to belong to each of the 4 classes for each ever smoker
#WeiTab=1 for each never smoker
```

```
#kt = 0 for controls, 1 for cases
#AgeIndexDate: age at index date
#depthab2: area of residence considered as factor variable with 38 as reference category
#ClTab=0 for never smokers, 1 for ever-smokers classified a posteriori in class 1, ... and 4 for ever-smokers classified a posteriori in class 1
#considered as factor variable with 0 as reference category



#logistic regression model 1: matching variables only
RegTabCrude<-glm(kt~I((AgeIndexDate/100)^-2)+I((AgeIndexDate/100)^-2 * log((AgeIndexDate/100)))+depthab2+ClTab,family=quasibinomial(),data=Base_All.Dup,weights=WeiTab)
summary(RegTabCrude)
logistic.display(RegTabCrude)

#logistic regression model 2: matching variables + ICE of occupational asbestos
RegTabCIE<-glm(kt~I((AgeIndexDate/100)^-2)+I((AgeIndexDate/100)^-2 * log((AgeIndexDate/100)))+depthab2+log(ICE_Am_M+0.1)+ClTab,family=quasibinomial(),data=Base_All.Dup,weights=WeiTab)
summary(RegTabCIE)
logistic.display(RegTabCIE)

#logistic regression model 3: matching variables + Variable of a posteriori classification for occupational asbestos
#ClAm: as factor variable with 0 as reference category of never exposed, 1 to 4 the posterior classfication and 5 for the very low exposed subjects
#WeiTabAm: the calculated probabilities for each combination among all the 5 classes of smoking and the 6 classes of asbestos

regLcmmClass<-glm(kt~I((AgeIndexDate/100)^-2)+I((AgeIndexDate/100)^-2 * log((AgeIndexDate/100)))+depthab2+ClAm+ClTab,family=quasibinomial(),data=Base_All.Dup,weights=WeiTabAsb)
logistic.display(regLcmmClass)


########################################
```
