# Supplementary material for: A new trajectory approach for investigating the association between an environmental or occupational exposure over lifetime and the risk of chronic disease: Application to smoking, asbestos, and lung cancer
Source: PLoS One. 2020 Aug 12;15(8):e0236736. doi: 10.1371/journal.pone.0236736 (PMC7423115; doi:10.1371/journal.pone.0236736)
Supplement: Attachment — Submitted filename: PONE.pdf [file pone.0236736.s002.pdf]

PONE-D-20-04889

This reviewer believes that authors undertook important effort to utilize statistical tools that are unjustly under-used in occupational epidemiology. Their approach should help derive more information from available data by getting away from bias that can arise from pre-specified time windows of exposure. It must be noted that both approaches have merit, especially if pre-specified time windows are infirmed by a priori hypotheses, thereby reducing bias that can arise in data- (rather than hypothesis-) driven analysis. This reviewer hopes that the authors see the merit of this critique of trajectory analyses and are willing to offer their opinion on this matter the introduction to the paper. But more importantly, this reader believes that the authors under-stated the importance of some of their etiological findings, which were enabled by novel method of analysis. (The authors discuss their etiological findings in light of prior research and note congruence, but it seems justified to be more assertive about the value of author's research to advancing policy-relevant knowledge.) Thus, this reviewer aimed his comments at strengthening the impact of the manuscript and anticipating challenges to validity of some of its more important conclusions to public health.

MAJOR COMMENTS

Table 2 implies that reducing smoking intensity confers massive health benefits despite considerable accumulated historical cig-years. This is a huge and very important finding!!! Cutting down on smoking is not futile!!! How do odds in these trajectories compare to not smoking at all? It seems important to know how odds in class 1 and 4 compare to abstinence. Please share these results: they may be one of the most important etiological and public health findings in your work and appear to arise due to trajectory analysis. Is it possible to repeat these analyses for "current smokers", such that the concerns about impact of ex-smokers (L212) are directly addressed? Please also adjust for trajectories of asbestos exposure, to explore vulnerability of this astonishing (in a good way) result to modeling choices.

Table 3, if one rightly puts p-values and test of null aside, does imply that class 4 (very distant high exposure intensity) is at the highest risk among exposed, with OR 1.3 [1.0, 1.8]. (NB: the second decimal place clearly carries no information here, especially in light of random error being insignificant compared to potential impact of unmodelled systematic errors due to measurement error, selection bias and latent confounding, see book by Lash et al). Class 3 tells similar story with OR that is between that of class 2 and 4, at 1.2 [0.9, 1.6]. Thus, focus on patterns in effect estimates tells a compelling story about importance of reduction of exposure to asbestos as early as possible in person's life. In other words, persons exposed to asbestos are not doomed to have lung cancer due to asbestos, but can improve their lot by reduction or elimination of exposure. This result also gives employers hope that reduction or elimination of exposure ASAP is beneficial. This pattern is the same as for smoking, bolstering its credibility. Weaker effects for asbestos were expected, but overall, the authors seem to have discovered support for something very important due to their methodological innovation. This reviewer urges the authors to be more proactive in stressing importance of their work beyond promotion of method well-used in other areas of scholarship. All comments on defending these analyses to challenges listed with respect to Table 2 also apply to Table 3.

One of the major concerns of this reviewer relates to the following choice by the authors, described on L110-111: What is the reason for these exclusions based on asbestos exposure? Being blow exposure projected under compliance with OEL is a weak argument: health effects often occur below OEL and it is critical to investigate health effects over full range of exposure in person's histories. Please present

results with and without these restrictions. The argument in discussion about conversion issues (L293+) comes rather too late and raise the question as to whether log(x+1) transformation (familiar to occupational health researchers, supported by theory and guaranteed to eliminate the troublesome zeros) is not be attempted in light of problems apparently arising from the use of relatively “exotic” I-spline transformation”. Please consider addressing is head-on, instead of leaving for future work. This seems simple to address in revisions!

L143-151: This reviewer finds it counter-intuitive that there are two sub-models 3: one with smoking as exposure and the other – with asbestos as exposure, each controlling for the other using different conceptualization of continuous exposures/trajectories. Is it not more consistent with the paper’s methodological intent to offer trajectories of smoking and exposure to asbestos in the same model? Segregation of one modeling aim: “what causes lung cancer” into two models that essentially consider the same exposures and hypothesis raises, needlessly, concerns about bias due to model-selection. Please consider replacing this modeling choice with a simpler one: only one sub-model 3.

Evaluation of the manuscript was limited by absence of Figures S1 and S2 among submitted materials. Please share.

This reviewer discourages authors from invoking “statistical significance” – it only hurts both the authors and the readers. Please see critique of p-values and “significance” culture; even statisticians are no longer willing to defend these approaches to epidemiology any more. See small sample of relevant arguments in

1: Greenland S. Null misinterpretation in statistical testing and its impact on health risk assessment. Prev Med. 2011 Oct;53(4-5):225-8. doi: 10.1016/j.ypmed.2011.08.010. Epub 2011 Aug 17. PubMed PMID: 21871481.

2: Greenland S, Senn SJ, Rothman KJ, Carlin JB, Poole C, Goodman SN, Altman DG. Statistical tests, P values, confidence intervals, and power: a guide to misinterpretations. Eur J Epidemiol. 2016 Apr;31(4):337-50. doi: 10.1007/s10654-016-0149-3. Epub 2016 May 21. PubMed PMID: 27209009; PubMed Central PMCID: PMC4877414.

3: Greenland S. Invited Commentary: The Need for Cognitive Science in Methodology. Am J Epidemiol. 2017 Sep 15;186(6):639-645. doi: 10.1093/aje/kwx259. PubMed PMID: 28938712.

How is one to judge that more information was extracted from data using LCMM? Examination of fit of the models and/or proportion of variability in outcome explained by the models? Compare to model fit when using formulation of smoking and asbestos exposures used in control for confounding by smoking/asbestos in table 2/3? This review would like to see calculations that address the question of gain in model fit/information due to trajectory analysis.

L228: Theoretical advances in statistics now allow us to assert that approaches like JEM lead to differential exposure misclassification when (a) exposure and outcome are known to be associated (as is the case with lung cancer and asbestos/smoking) and (b) inherently continuous exposure (like smoking and asbestos) are categorized. Please see Appendix A of Singer et al [https://www.ncbi.nlm.nih.gov/pubmed/30030714] for accessible form of the relevant arguments derived from textbook on measurement error by Prof P Gustafson. Ref 35 is outdated, deals with a special case and does not reflect modern understanding of the measurement error problem.

SECONDARY MATTERS RELATIVE TO MAJOR COMMENTS

The article can benefit from language editing, but this reviewer has not major concerns, other than needless vagueness introduced by use of undefined adjectives, like “often”, “generally”, “few”, “recent”, “actual”, “rarely”, “much stronger”, etc. The authors may wish to sharpen their arguments by weeding our such distractions.

L45: the problem is not particular to study of cancers.

L46-77: This reviewer is unsure by that is meant by reference to “two months” in “… incidence density sampling every two months”. Please clarify.

L78: This reviewer is unsure about what is meant by stratification on SES. Did the authors implement frequency matching of cases and referents on SES within sex-age-residence strata? Please clarify early on presentation of methods, such that the mention of matching later on in the narrative does not surprise readers.

L85: Please include copy of questionnaire and interview guide as supplemental materials.

L102: Please give equation used to “prorate” mean level of exposure by duration of each job within a year. Was person exposed to X for 10 months assigned 10X/12 mean exposure in that year, a cumulative within-year exposure metric?

L108: Please clarify what is meant by “were yet”.

L108-109: How many subjects were excluded due to missing data and how do they compare to those retained in analysis? This is explained for asbestos in Table S2 but other exclusions.

L130-132: Exposure intensity is typically assumed to have log-normal distributions with log(X+1) transformation typically being sufficient, including accounting for zero exposure. There are strong theoretical reasons for log-normality. Why did the authors deviate from this practice? What impact did it have on the results? It would greatly help acceptance of the proposed methodological innovation if it was limited to trajectory analysis, leaving other matters more familiar/accepted. Otherwise, a skeptical reader would know whether different results were due to modeling of trajectories or other analytical choices, like restriction to asbestos exposures >OEL or exotic transformation of exposure levels. AIC is not the key argument here, especially if not shown for log-transformation (and this reviewer did not see any AIC in the paper AND S1 Fig is not among submitted materials). THIS IS ALSO ADDRESSED AMONG MAJOR COMMENTS AND IS REALLY A RATHER EASY ISSUE TO ADDRESS.

L139-142: What was assumed about covariance structure of random effects?

L159-160: It is reasonable to provide annotated computing code without additional requests from readers: request from this reviewer should suffice. Please share your annotated code in supplemental materials.

Figures 2 and 3 are excellent!!! Thank you. Only minor suggestion is to consider re-drawing using colorblind palette of R, to help some (mostly male) readers who are at least partially colorblind.
