# Supplementary material for: A new trajectory approach for investigating the association between an environmental or occupational exposure over lifetime and the risk of chronic disease: Application to smoking, asbestos, and lung cancer
Source: PLoS One. 2020 Aug 12;15(8):e0236736. doi: 10.1371/journal.pone.0236736 (PMC7423115; doi:10.1371/journal.pone.0236736)
Supplement: Attachment — Submitted filename: Response_to_Reviewers.pdf [file pone.0236736.s004.pdf]

Response to Reviewer 1

We thank the reviewer for careful and in-depth revision of our manuscript, and for very insightful and useful suggestions that helped us to significantly improve our manuscript as described below.

P1. This reviewer believes that authors undertook important effort to utilize statistical tools that are unjustly under-used in occupational epidemiology. Their approach should help derive more information from available data by getting away from bias that can arise from pre-specified time windows of exposure. It must be noted that both approaches have merit, especially if pre-specified time windows are infirmed by a priori hypotheses, thereby reducing bias that can arise in data- (rather than hypothesis-) driven analysis. This reviewer hopes that the authors see the merit of this critique of trajectory analyses and are willing to offer their opinion on this matter the introduction to the paper. But more importantly, this reader believes that the authors under-stated the importance of some of their etiological findings, which were enabled by novel method of analysis. (The authors discuss their etiological findings in light of prior research and note congruence, but it seems justified to be more assertive about the value of author's research to advancing policy-relevant knowledge.) Thus, this reviewer aimed his comments at strengthening the impact of the manuscript and anticipating challenges to validity of some of its more important conclusions to public health.

As suggested, we have clarified in the introduction that the proposed trajectory approach may provide an alternative data-driven analytical approach compared analyses based on pre-specified time windows of exposure. We further clarified in the title of the paper that the proposed approach is a trajectory approach. We also strengthen our etiological findings, as described in our responses to each specific point below.

MAJOR COMMENTS

P2. Table 2 implies that reducing smoking intensity confers massive health benefits despite considerable accumulated historical cig-years. This is a huge and very important finding!!! Cutting down on smoking is not futile!!! How do odds in these trajectories compare to not smoking at all? It seems important to know how odds in class 1 and 4 compare to abstinence. Please share these results: they may be one of the most important etiological and public health findings in your work and appear to arise due to trajectory analysis. Is it possible to repeat these analyses for "current smokers", such that the concerns about impact of ex-smokers (L212) are directly addressed? Please also adjust for trajectories of asbestos exposure, to explore vulnerability of this astonishing (in a good way) result to modeling choices. Table 3, if one rightly puts p-values and test of null aside, does imply that class 4 (very distant high exposure intensity) is at the highest risk among exposed, with OR 1.3 [1.0, 1.8]. (NB: the second decimal place clearly carries no information here, especially in light of random error being insignificant compared to potential impact of unmodelled systematic errors due to measurement error, selection bias and latent confounding, see book by Lash et al). Class 3 tells similar story with OR that is between that of class 2 and 4, at 1.2 [0.9, 1.6]. Thus, focus on patterns in effect estimates tells a compelling story about importance of reduction of exposure to asbestos as early as possible in person's life. In other words, persons exposed to asbestos are not doomed to have lung cancer due to asbestos, but can improve their lot by reduction or elimination of exposure. This result also gives employers hope that reduction or elimination of exposure ASAP is beneficial. This pattern is the same as for smoking, bolstering its credibility. Weaker effects for asbestos were expected, but overall, the authors seem to have discovered support for something very important due to their methodological innovation. This reviewer urges the authors to be more proactive in stressing importance of their work beyond promotion of method well-used in other areas of scholarship. All comments on defending these analyses to challenges listed with respect to Table 2 also apply to Table 3.

We thank the reviewer for this major comment. As suggested:

- We have compared the odds of lung cancer of each class of exposure trajectories to the class of never exposed, for both smoking (new Table 2) and asbestos (new Table 3). Never exposed subjects are thus now also included in the new Table 1 which describes the study population. Please also note that to compare the odds to never smokers, we had to use a two-stage analysis. In stage 1, profiles of exposure trajectories were identified among ever exposed subjects with a latent class mixed model (a LCMM made of “Sub-model 1” and “Sub-model 2” as in the original submission). In stage 2, the odds of lung cancer of identified profiles were compared to the odds of never exposed with a logistic regression (“Sub-model 3” in the original submission). In the original submission, the three sub-models were estimated in a single stage with a joint LCMM. The results presented in the revised manuscript are thus now not exactly the same as in the original submission, although very similar.
- We added a secondary analysis on current smokers only (new Fig S3, new Table S5 in Supplementary materials) to address the concern by ex-smokers and time since smoking cessation, which further allowed us to better distinguish the effect of intensity from duration.
- We clarified major etiological findings and their public health implications (second paragraph of the Discussion).
- We removed the second decimal place for all ORs (new Tables 2, 3, and S5).

P3. One of the major concerns of this reviewer relates to the following choice by the authors, described on L110-111: What is the reason for these exclusions based on asbestos exposure? Being blow exposure projected under compliance with OEL is a weak argument: health effects often occur below OEL and it is critical to investigate health effects over full range of exposure in person’s histories. Please present results with and without these restrictions. The argument in discussion about conversion issues (L293+) comes rather too late and raise the question as to whether log(x+1) transformation (familiar to occupational health researchers, supported by theory and guaranteed to eliminate the troublesome zeros) is not be attempted in light of problems apparently arising from the use of relatively “exotic” I-spline transformation”. Please consider addressing is head-on, instead of leaving for future work. This seems simple to address in revisions!

We agree with the reviewer that the issue of convergence came too late, and we apologize for the absence of the figure showing the distributions of asbestos annual intensities in the Supplementary materials (now in new Fig S1). We also agree that the log transformation may be used for the cumulative dose of asbestos exposure (CIE). Fractional polynomials indeed indicated this was the best parametric transformation of CIE (new Table 2, footnote g). However, for annuals intensities of asbestos exposure, the distribution was much more skewed (new Fig S1b, Supplementary materials), and the log transformation did not allow convergence of LCMM estimates, whether excluding or not the low-exposure subjects. To achieve convergence, we had to use a more flexible normalizing transformation. For that purpose, we used the I-spline function implemented in the lcmm R package which allows normalizing distributions that are far from the normal distribution, without imposing strong assumptions. Please note that even with that flexible function, we still had to exclude the low-exposed subjects from the LCMM analysis to achieve convergence because these subjects induced a too strong peak of annual intensities at zero due to years without any occupational exposure to asbestos. This has been clarified in the statistical analysis sub-section (paragraph 4). More importantly; to strengthen our etiological findings as suggested, we have included all these very low exposed subjects in an a priori class of exposure, described exposure within this class (new Fig 3 and Table 3), and compared their odds of lung cancer to the odds of never exposed (new Table 3).

P4. L143-151: This reviewer finds it counter-intuitive that there are two sub-models 3: one with smoking as exposure and the other – with asbestos as exposure, each controlling for the other using different conceptualization of continuous exposures/trajectories. Is it not more consistent with the paper's methodological intent to offer trajectories of smoking and exposure to asbestos in the same model? Segregation of one modeling aim: "what causes lung cancer" into two models that essentially consider the same exposures and hypothesis raises, needlessly, concerns about bias due to model-selection. Please consider replacing this modeling choice with a simpler one: only one sub-model 3.

We thank the reviewer for this important comment. We agree with the reviewer that the choice of adjustment method is important, and it may appear counter-intuitive to use different modelling strategies depending on the role played by the substance in the model (exposure or confounder). Therefore, as suggested, in the revised manuscript, we have mutually adjusted the identified profiles of smoking and asbestos exposure trajectories, using only one logistic regression model. Please note that we used a two-stage approach as mentioned in our response to Point P2 above. In stage 1, the profiles (i.e. latent class) of smoking and asbestos exposure trajectories were identified using two separate LCMMs, one for smoking and one for asbestos, since identification of profiles does not need to be adjusted for any potential confounders. In stage 2, the association between class membership and the risk of lung cancer was estimated using a weighted logistic regression model including both the classification for smoking and the classification for asbestos, as suggested by the reviewer (the results are shown in the last columns of new Table 2 and Table 3). In stage 2, subjects were weighted according to their posterior probability to belong each class (derived from the LCMM), in order to account for uncertainty of classification resulting from stage 1, as now explained in the revised manuscript (second last paragraph of the Statistical Analysis sub-section). However, while we agree that it may appear counter-intuitive to use different modelling strategies depending on the role played by the substance in the model (exposure or confounder), we still believe that it is not straightforward that adjusting for the class of trajectories of the confounder is better than adjusting for its cumulative dose at the index date. For comparison purpose and to assess the robustness of results with respect to the choice of modelling for the confounder, we decided to show the results for three sets of adjustment: no adjustment for the confounder, adjustment for the cumulative dose of the confounder at the index date, and adjustment for the class of exposure trajectories of the confounder (new Tables 2 and 3). We compared the results in terms of estimated OR, as well as in terms of AIC to also respond to Point 7 below. As expected, estimations of OR were more sensitive to the modelling of smoking for asbestos exposure than to the modelling of asbestos for smoking, likely because of the strong impact of smoking on lung cancer. We further attempted to discuss and explain the divergence of results for asbestos, based on the observed association between the identified classes of trajectories for smoking and asbestos exposure (new Table S6, Supplementary materials). The Methods, Results, and Discussion sections have all been modified accordingly. We hope the reviewer will find these new results of methodological and etiological interest.

P5. Evaluation of the manuscript was limited by absence of Figures S1 and S2 among submitted materials. Please share.

We apologize for the absence of Figures S1 and S2 in the submitted materials of the first submission, likely due to a bug during the submission process. To avoid this issue in the current submission, we have merged all the supplementary materials in a single file where you will now find Figures S1 and S2.

P6. This reviewer discourages authors from invoking "**statistical significance"** – it only hurts both the authors and the readers. Please see critique of p-values and "significance" culture; even statisticians are no longer willing to defend these approaches to epidemiology any more. See small sample of relevant arguments in 1: Greenland S. Null misinterpretation in statistical testing and its impact on health risk assessment. Prev Med. 2011 Oct;53(4-5):225-8. doi: 10.1016/j.ypmed.2011.08.010. Epub 2011 Aug 17. PubMed PMID: 21871481. 2: Greenland S, Senn SJ, Rothman KJ, Carlin JB, Poole C, Goodman SN, Altman DG. Statistical tests, P values, confidence intervals, and power: a guide to misinterpretations. Eur J Epidemiol. 2016 Apr;31(4):337-50. doi: 10.1007/s10654-016-0149-3. Epub 2016 May 21. PubMed PMID: 27209009; PubMed Central PMCID: PMC4877414. 3: Greenland S. Invited Commentary: The Need for Cognitive Science in Methodology. Am J Epidemiol. 2017 Sep 15;186(6):639-645. doi: 10.1093/aje/kwx259. PubMed PMID: 28938712.

We fully agree that the binary interpretation of quantitative p-values or confidence intervals is an important issue in health literature, so as suggested, we removed the few terms invoking statistical significance.

P7. How is one to judge that more information was extracted from data using LCMM? Examination of fit of the models and/or proportion of variability in outcome explained by the models? Compare to model fit when using formulation of smoking and asbestos exposures used in control for confounding by smoking/asbestos in table 2/3? This review would like to see calculations that address the question of gain in model fit/information due to trajectory analysis.

We understand that the reviewer suggests to compare the model fit when adjusting for the cumulative dose of the confounder (as in the original submission) or for its classification resulting from the LCMM (as now performed following suggestions in Point 4 above). As suggested and as mentioned in Point P4 above, the AIC corresponding to the two modelling are now showed in the last rows of new Tables 2 and 3. The results show that adjusting for the quantitative cumulative dose at the index date provided a better fit to data than classification of the confounder trajectories. This is particularly true for the CSI which provided a much better fit than classes of smoking trajectories. This point is further discussed in the revised manuscript (second paragraph of the Discussion). Please also note that while we agree that model fit may help selecting the best strategy for the modelling of the confounder, we don't think it should be the most important criteria to choose between different types of modelling of the exposure in an etiological study. Indeed, the reviewer probably agrees that the type of modelling chosen for the exposure should depend above all on the research question. If the research question is about exposure trajectories over lifetime and their association with the risk of disease, the standard cumulative dose of exposure cannot be an option.

P8. L228: Theoretical advances in statistics now allow us to assert that approaches like JEM lead to differential exposure misclassification when (a) exposure and outcome are known to be associated (as is the case with lung cancer and asbestos/smoking) and (b) inherently continuous exposure (like smoking and asbestos) are categorized. Please see Appendix A of Singer et al [https://www.ncbi.nlm.nih.gov/pubmed/30030714] for accessible form of the relevant arguments derived from textbook on measurement error by Prof P Gustafson. Ref 35 is outdated, deals with a special case and does not reflect modern understanding of the measurement error problem.

Asbestos exposure was not categorized in a standard way in our study since annual intensities derived from the JEM were quantitatively modelled using a mixed model including random effects and random measurement errors. The classification was made on trajectories of intensities over time, and not as

usual on the cumulative dose of exposure assuming no measurement errors at all. However, we acknowledge that this point had to be clarified in the manuscript, where we have now included the reference to Gustafson's textbook and Singer et al's paper (Discussion, first paragraph of limitations of our study).

SECONDARY MATTERS RELATIVE TO MAJOR COMMENTS

P9. The article can benefit from language editing, but this reviewer has not major concerns, other than needless vagueness introduced by use of undefined adjectives, like "often", "generally", "few", "recent", "actual", "rarely", "much stronger", etc. The authors may wish to sharpen their arguments by weeding our such distractions.

We removed these terms when not necessary.

P10. L45: the problem is not particular to study of cancers.

We replaced "cancer" by "chronic disease" everywhere when applicable in the title, abstract, introduction and discussion.

P11. L46-77: This reviewer is unsure by that is meant by reference to "two months" in "… incidence density sampling every two months". Please clarify.

P12. L78: This reviewer is unsure about what is meant by stratification on SES. Did the authors implement frequency matching of cases and referents on SES within sex-age-residence strata? Please clarify early on presentation of methods, such that the mention of matching later on in the narrative does not surprise readers.

As requested, we clarified the selection of controls in the revised manuscript (Study design subsection).

P13. L85: Please include copy of questionnaire and interview guide as supplemental materials.

We understand the request of the reviewer, but the questionnaire is in French and would therefore be of limited interest for most readers, and the PIs of the ICARE study do not wish to make their questionnaire publicly available.

P14. L102: Please give equation used to "prorate" mean level of exposure by duration of each job within a year. Was person exposed to X for 10 months assigned 10X/12 mean exposure in that year, a cumulative within-year exposure metric?

We apologize for this sentence which induced confusion. We actually did not prorate by the duration of each job held in the calendar year, but just derived the unweighted average of the level of exposure of all jobs held in the year. This has been clarified in the manuscript with a concrete example of derivation (end of sub-section on Occupational asbestos exposure assessment).

P15. L108: Please clarify what is meant by “were yet”.

This part has been largely reformulated because of the new adjustment method as requested in the major point P4 above, and this sentence has been removed.

P16. L108-109: How many subjects were excluded due to missing data and how do they compare to those retained in analysis? This is explained for asbestos in Table S2 but other exclusions.

As shown in Fig 1, 250 cases and 170 controls were excluded because of missing information on smoking and/or job history. New Table S2 (Supplementary materials) now compares their characteristics to the 2026 cases and 2610 controls retained in the analysis. All this has been clarified the first paragraph of the results section.

P17. L130-132: Exposure intensity is typically assumed to have log-normal distributions with log(X+1) transformation typically being sufficient, including accounting for zero exposure. There are strong theoretical reasons for log-normality. Why did the authors deviate from this practice? What impact did it have on the results? It would greatly help acceptance of the proposed methodological innovation if it was limited to trajectory analysis, leaving other matters more familiar/accepted. Otherwise, a skeptical reader would know whether different results were due to modeling of trajectories or other analytical choices, like restriction to asbestos exposures >OEL or exotic transformation of exposure levels. AIC is not the key argument here, especially if not shown for log-transformation (and this reviewer did not see any AIC in the paper AND S1 Fig is not among submitted materials). THIS IS ALSO ADDRESSED AMONG MAJOR COMMENTS AND IS REALLY A RATHER EASY ISSUE TO ADDRESS.

Please see our response to Point P3 above, as well as Point P7 for AIC.

P18. L139-142: What was assumed about covariance structure of random effects?

We used a random effect on the intercept only, and not on regression coefficients of the spline function of time. There was therefore no need to specify any structure for the covariance matrix of random effects. This has been clarified in the paragraph 5 of the Statistical Analysis subsection.

P19. L159-160: It is reasonable to provide annotated computing code without additional requests from readers: request from this reviewer should suffice. Please share your annotated code in supplemental materials.

We included the annotated R code in Supplementary materials as requested.

P20. Figures 2 and 3 are excellent!!! Thank you. Only minor suggestion is to consider re-drawing using colorblind palette of R, to help some (mostly male) readers who are at least partially colorblind.

We thank the Reviewer for his enthusiastic comment! We used a colorblind-friendly palette of R (viridis package) as suggested.
